# Comparison of gesture-vocal synchrony and gestures of two children aged 3 to 26 months with and without autism spectrum disorder

**DOI:** 10.1590/2317-1782/e20240311en

**Published:** 2025-10-27

**Authors:** Gabriela Luisa Gantier Fernández, Marianne Carvalho Bezerra Cavalcante, Ana Paula Ramos de Souza

**Affiliations:** 1 Universidade Federal de Santa Maria – UFSM – Santa Maria (RS), Brasil.; 2 Programa de Pós-graduação em Letras, Universidade Federal da Paraíba – UFPB – João Pessoa (PB), Brasil.; 3 Departamento de Saúde e Comunicação Humana, Universidade Federal do Rio Grande do Sul – UFRGS - Porto Alegre (RS), Brasil.; 4 Programa de Pós-graduação em Distúrbios da Comunicação Humana – PPGDCH, Universidade Federal de Santa Maria – UFSM - Santa Maria (RS), Brasil.

**Keywords:** Autism, Speech, Multimodality, Language, Child Development

## Abstract

The objective was to compare gesture-vocal synchrony in language functioning between mothers and babies and the gestural typology of babies from 3 to 26 months of age, one of them with autism spectrum disorder (case R), diagnosed at age 3, and the other without diagnosis (case B). It was select moments in which there was greater mother-baby interaction, from a bank of mother-baby interactions footage, from 3 to 26 months. It was analyzed using the Eudico Linguistic Annotator software ( ELAN), considering multimodal categories of sign language and speech of mothers and babies for descriptive statistical analysis. The results showed differences in the frequencies and types of gestures between the babies and also in the synchrony between them and their mothers. In the case of B., the gesture-vocal synchrony and variety of gestures is inserted in a context of conjunction between him and his mother in the first year of life, which gave rise to the second enunciative mechanism in the second year of life. In case R, the conjunction relationships were not established, as the difficulties in reading discomfort and annoyance gestures, very present in the baby since its first months of life, hindered the engagement and shared attention of the mother and her baby, disfavoring the inscription of gesture as language. R. showed language acquisition delay at 26 months. Although the gestural typology of B and R are similar, the frequencies, the quality and, above all, the gesture-vocal synchrony differ between dyads.

## INTRODUCTION

Gesture-speech synchrony has been studied in understanding the emergence of childhood fluency^(1,2)^, highlighting, based on classic studies on human gestures, that there is continuity and integration between gesture and speech in the functioning of children's language. In this sense, synchrony occurs when the modalities of language use (speech and gesture) work together in linguistic production, creating points of salience in the utterance. This is an approach that offers contributions to thinking about atypical language development^(3)^.

In this study, this multimodal approach to linguistic interactions is incorporated into the enunciative perspective of language acquisition proposed by Silva^(4)^, in order to reflect on the initial conjunction and disjunction relations between mother and baby, permeated by processes of homology and interpretance in the relationship between the baby's gestural and vocal manifestations and the mother's verbal ones, present in the early protoconversations^(5)^.

It is known that the transition from the baby's discursive dependence on the mother, present in conjunctive relations, to the recognition of the effects of their manifestations on the other, present in disjunctive relations, propels babies towards the constitution of the second enunciative mechanism, which is the shift from shown reference to spoken reference^(4)^. For this transition to occur, the baby must be able to occupy their place of enunciation, and the mother must sustain this place for them^(6)^.

In this sense, a study^(7)^ highlighted both difficulties in the exercise of parental functions, in the supposition of the subject, and in the establishment of demand^(7)^, as well as the risk of evolution towards an ASD (Autism Spectrum Disorder) condition^(7)^. Oliveira et al.^(8)^ observed a higher correlation between patterns of psychic suffering in the first six months of life. In the second, third, and fourth semesters of the babies' lives, psychic suffering and language acquisition delays coincide, but there are also cases of language acquisition delay without psychic risk. These studies showed that the presence of psychic suffering, whether or not it is moving towards an autistic structure, is significantly correlated with the presence of language acquisition delay, analyzed from an enunciative perspective.

The evaluation method in both studies^(7,8)^ used the Enunciative Signs of Language Acquisition^(9,10)^, which analyze the conjunctive and disjunctive relations of the first enunciative mechanism as well as the emergence of the second enunciative mechanism, but without a more detailed discrimination of gestural aspects. Therefore, in this research, the aim was not only to investigate the enunciative effects of psychic suffering but also to add a multimodal perspective to the study of the language acquisition process, especially regarding the first and second enunciative mechanisms^(4)^.

This is based on the belief that investigating the multimodal characteristics of gesture-speech synchrony—such as speech, gesture, directed gaze, facial expressions, head movements, and other indicators—can shed light on signs for the early detection of risk and for timely intervention that seeks to establish mother-baby enunciative conjunction relations, which form the foundation for the emergence of subsequent enunciative mechanisms in the language acquisition process.

Considering the group of children with language acquisition delay, although there are already studies on gesture-speech synchrony in children with Down syndrome^(3)^, no studies have been found addressing this synchrony in children with ASD within the national context, integrated with the enunciative perspective. Thus, the objectives of this study are to compare gesture-speech synchrony in language functioning between mothers and babies, as well as the gestural typology of babies aged 3 to 26 months, one of whom has autism spectrum disorder (Case R), diagnosed at the age of 3, and the other without this diagnosis (Case B).

## METHOD

This is a qualitative, longitudinal, observational, analytical, and comparative case study;involving two children, one with ASD (R) and one without (B), followed from 3 months to 26 months of age. The study was approved by the university's research ethics committee under CAAE number 28586914.0.0000.5346, opinion number 1.929.266. The children's legal guardians signed the informed consent form, authorizing their participation in the study.

The video data of the interactions between the babies and their mothers analyzed in this study come from a larger database in which the babies were filmed across six age ranges for 15 minutes each. In addition, the babies and their mothers were assessed using the Enunciative Signs of Language Acquisition (SEAL)^(9,10)^, the Clinical Indicators of Risk/Reference^^*^^ for Child Development (IRDI)^(11)^, PREAUT Signs^(12)^, M-CHAT^(13)^, and Bayley Scales of Infant and Toddler Development, Third Edition (Bayley-III)^(14)^. The following collection and assessment procedures were applied for each age range:

- Age Range 1 – 3 months and 1 day to 4 months and 29 days: In a seated position in an infant car seat (9 minutes). In this position, the mother was invited to sing (3 minutes), talk (3 minutes), and offer an object to the baby—a silent rubber dog (3 minutes). In prone (3 minutes) and supine (3 minutes) positions, the mother could optionally offer a toy or talk to the baby. Assessed with IRDI Range I, PREAUT Signs, SEAL.
- Age Range 2 – 5 months and 1 day to 6 months and 29 days**:** In a seated position in an infant car seat (9 minutes). The mother was invited to sing (3 minutes), talk (3 minutes), and offer the rubber dog to the baby (3 minutes). In prone (3 minutes) and supine (3 minutes) positions, the mother could offer a toy or engage in conversation. Assessed with SEAL.
- Age Range 3 – 8 months and 1 day to 9 months and 29 days**:** Seated without support if possible (9 minutes). The mother was instructed to sing to the baby (3 minutes), talk (3 minutes), and offer the rubber dog (3 minutes). In prone (3 minutes) and supine (3 minutes) positions, the mother was asked to stimulate the baby to roll over or crawl, and attempts by the baby to stand with or without support were observed. Assessed with IRDI Phase II, PREAUT Signs, SEAL.
- Age Range 4 – 11 months and 1 day to 12 months and 29 days**:** In this stage, the baby was free to explore a box of themed toys (transportation, play food, dolls, animals) with the mother on an EVA foam mat. The baby could sit or walk as long as they stayed on the mat for filming purposes. Ten minutes were spent with the mother and five minutes with the therapist entering the scene to check some enunciative signs. Assessed with IRDI Phase III, SEAL.
- Age Range 5 – 17 months and 1 day to 18 months and 29 days: Same procedure as Range 4. Assessed with IRDI Phase IV, SEAL, Bayley-III, M-CHAT.
- Age Range 6 – 22 months and 1 day to 26 months: Same procedure as Ranges 4 and 5. Assessed with SEAL, Bayley-III, M-CHAT.

From the video database, which included moments when the mothers sang, talked, and played with the children, excerpts were selected in which face-to-face interactions were more prominent, standardizing both the duration and conditions between the two cases, as shown in Table 1.

**Table 1:** Description of age groups and filming analysis time per subject

Nº	Age range	Subject	Age of child At collection	Temporal composition of samples	
				Original time of video	Sample selected for ELAN analysis
**1**	3m 1d – 4m 29d.	R	3 m 25d	18min25 sec	2 min 44 sec
		B	3m7d	14min 19 sec	3 min 31 sec
**2**	5m 1d – 7m 29d	R	6 m 7d.	19 min. 35 sec	2 min 42sec
		B	6m 6d	21 min 51 sec	2 min 32 sec
**3**	8m 1d- 9 m 29 d	R	8 m 8 d	16min 1sec	2min 33 sec
		B	9m 29d	12 min 31 sec	2 min 22 sec
**4**	11m 1d- 12m 29d	R	12m 17d	17 min 17sec	2min 40 sec
		B	12m13d	16min 58 sec	2 min 38 sec
**5**	17m 1d – 18m 29 d.	R	17 m.	19min 34 sec	2 min 43 sec
		B	17m 19d	13 min 9 sec	2 min 47 sec
**6**	22m 1d – 26 m 29d	R	26 m 20 d	16min 3 sec	2min. 53 sec
		B	22m	14 min 29 sec	2min 31 sec

m=months d=days min=minutes sec=seconds

It is important to highlight that once risk signs were identified in R. during the initial assessments, efforts were made to provide timely intervention; however, the child remained in therapy for only two months, from 10 to 12 months of age. This had an impact during Phase 5 but was not sustained over time, as by 26 months—his last recording—he exhibited clear signs of ASD.

Based on this selection, the data were analyzed using **Eudico Linguistic Annotator (ELAN)** software, which enabled the transcription of excerpts from key interaction moments for detailed visualization, time coding, and descriptions according to the established tracks, as described in Table 2.

**Table 2:** Standards of multimodal transcriptions

Tracks	Symbols
Mother’s speech	-.......................-
Mother’s silence	*.......................*
Mother's head movement	/......................./
Mother’s gaze	(.......................)
Mother's hand movements	_......................._
Mother's gestures	+.......................+
Child’s speech	--.......................--
Child’s silence	**.......................**
Child’s head movements	//.......................//
Child’s gaze	((.......................))
Child’s hand movements	__......................__
Child’s gestures	++......................++

The study also aimed to identify the emerging gestural typology in each case at each age range, as described in Table 3.

**Table 3:** Gesture typology

Vocal Pauses	Silences longer than 7 seconds were considered vocal pauses.
Gesture typology	The body gesture typology was analyzed, considering emblems, pantomimes, gestures of discomfort, gestures of happiness, filling gestures, among others that emerged
Eye Contact	The eye contact was analyzed in seconds, and the addressing of the mother and child was also analyzed.
Exchange Times	The times of synchronous exchange in the dyads were marked.
Mother's' Touch	The mothers' touch belongs to the deictic gestures and sometimes plays a role as fillers within the typology, but it was analyzed separately from the typology for better sensory understanding in the cases.

From the ELAN annotations, descriptive statistics were generated and are presented in the Results section.

## RESULTS

Table 4 presents the results of the general categories of the analyses.

**Table 4:** General description of results

Age range	Cases	Mother’s Silences	Mother’s Gestures	Mother’s Speech	Mother’s gaze	Child’s silences	Child’s Speech	Dyad’s Eye contact	Child’s gesture-vocal synchrony
1	R	0	9	60	31	6	24	6	7
	B	0	27	63	23	5	49	10	8
2	R	0	15	54	17	6	5	3	0
	B	0	4	52	33	4	27	11	4
3	R	0	26	59	14	4	18	3	12
	B	0	13	48	9	4	31	6	12
4	R	0	27	50	15	8	17	5	2
	B	0	20	77	17	9	26	11	7
5	R	2	11	54	27	6	11	4	5
	B	0	15	60	27	2	39	12	26
6	R	0	8	69	30	8	15	1	8
	B	0	21	48	36	1	32	15	20

**Figure 1:**
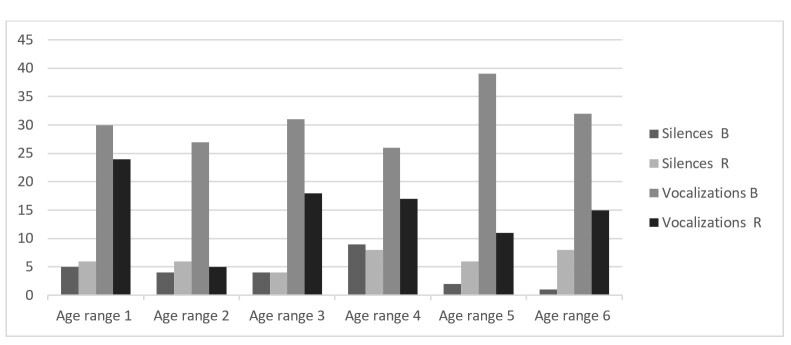
Analysis of the evolution of silences and vocalizations

**Figure 2:**
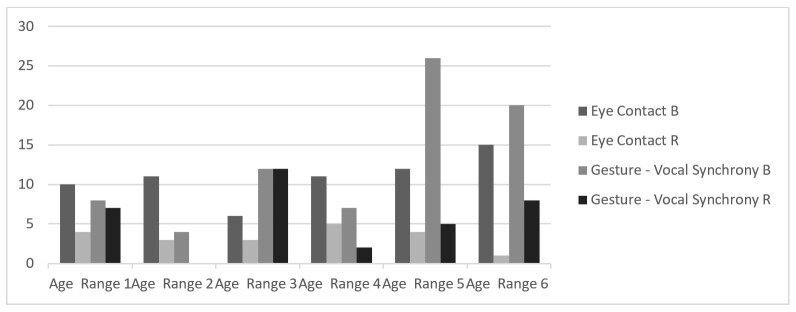
Analysis of the evolution of eye contact and gesture-vocalization synchrony

These results show that there are no significant distinctions between the mothers. However, among the children, clearer distinctions emerge in eye contact, child vocalization, and gesture-speech synchrony, with Case R consistently showing lower values. These differences become more visually evident in Figures 1 and 2.

Thus, while B progressively expands vocalizations towards speech, gesture-speech synchrony, and eye contact with the mother—reducing silence—R maintains lower levels of vocalization and gesture-speech synchrony, decreases eye contact with his mother, and increases periods of silence. In this way, it can be said that while dyad B consolidates language functioning through dialogue, dyad R does not evolve as expected over the course of the follow-up.

**Figure 3:**
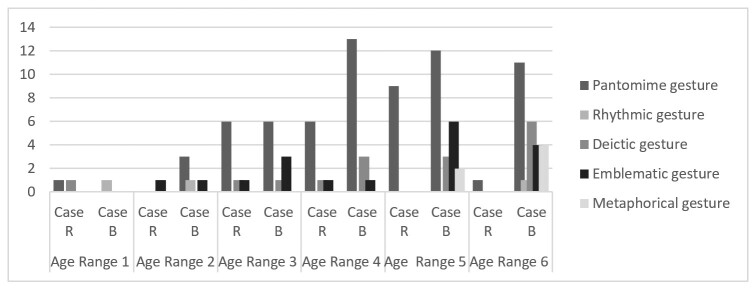
Gestural development of the babies

Regarding gestural development, Table 5 presents the general expressions, and Figure 3 shows the gestural typology of each child, based on the initial gesture classification used in the study.

**Table 5:** Evolution of babies' general expressions

expression	R1	B1	R2	B2	R3	B3	R4	B4	R5	B5	R6	B6
Smile/happiness	0	6	0	10	8	8	4	0	0	1	6	1
complaint/nervous	3	0	0	0	0	0	1	0	0	0	0	0
Uncomfortable/discomfort	2	0	3	0	9	0	2	0	1	0	2	0
scare/defense	3	0	0	0	0	0	0	0	2	0	0	0
sadness	0	0	2	0	1	0	0	0	0	0	0	0

1, 2, 3, 4, 5, 6= number of age range R and B = Subjects

In R's case, there were general facial expressions (Table 5), displaying gestures of complaint, discomfort, surprise, or nervousness that differed from B’s from the very first phase. In age range 2, complaints and discomfort dominated his manifestations. In age ranges 3 and 4, in addition to gestures of complaint, discomfort, and unease, gestures of happiness (Table 5), pantomime, deictic gestures, and emblems (Figure 3) were added. This shows that he may present a gesture typology similar to B in the expected variety of gestures but differs in the constant presence of discomfort, unease, and complaint. Another difference lies in gesture quality: R’s pantomime was always the same—reproducing a “vroom-vroom” for the toy car—demonstrating his hyperfocus on the car, while B’s pantomimes varied, covering different animals, vehicles, and feeding scenes.

Multimodal transcription examples from age phases 1 and 6 for both cases are presented in Chart 1.

**Chart 1:** examples of multimodal transcriptions of B and R

Multimodal transcriptions	
**Age range 1- case R**	**Age range 1- case B**
MR- Come on and play a little, are you lazy?	MB-nhammmm-
(she looks at his foot, look at his leg )	/ Leave B.'s head in a firm position /
(she looks at the child)	_ the mother takes her hands back and makes movements in the air _
	+ the mother makes a pantomime gesture of a walking spider +
	(the mother looks at the child's feet)
R// he turnr your head to the left //	B-- Vocalization with vowel aaaa--
((He looks at another point on the left ))	((B looks at his mother smiling and enjoying the contact))
	
MR_ Lower her hands _ (she looks down)	MB- I'm going to pick up the little baby -
- where's the baby screaming ahh?	_ she picks up the child's feet and caresses them _(she looks at the child's face)
(she looks at the child )	
**Age range 6- case R**	**Age range 6- case B**
R((he looks at the other toys on the floor))	B --“gr ahhhh u gr ahh u” dinosaur onomatopoeia --
++ he makes a gesture of pain reaction ++	++ pantomime gesture of dinosaur attacking++
Child speech-gesture synchrony__ he touches the floor with his left hand_	Gesture-vocal synchrony
MR_ with the other hand, she presents the toy and gives it to the child__	MB_ she makes the doll swing on the floor _
R((he closes his eyes))	B__ he quickly moves dinosaur in the air __
	((he looks at the toys on the floor))

R: case R, MR: mother of R, Case B, MB: mother of B

As shown in Chart 1, R demonstrates more discomfort and less progress in shared attention and engagement with his mother compared to B. While B shows pleasure in physical contact, R avoids contact with his mother in age range 1. In age range 6, B dramatizes a dinosaur attack on a doll with his mother using pantomime, while R barely engages in his mother's play proposals.

It is important to highlight that during R’s moments of discomfort, it was common for the mother to interpret his reactions as anger rather than as signs of pain. Over the two years of follow-up, the mother was unable to perceive that R experienced physical pain linked to sensory issues, including tactile, auditory, visual, and vestibular hypersensitivity, among other difficulties.

## DISCUSSION

From the very first phase, the difference between R and B is clear: in the multimodal transcriptions, R exhibits less varied gestures, with lower frequency and shorter duration. Notably, there is less synchrony between speech and gesture, as well as reduced eye contact, as shown in Figure 2. In addition, R produces fewer vocalizations than B and has more observable moments of silence (Figure 1). There are many instances of discomfort and unease in R, while B is consistently smiling and enjoying physical, visual, and bodily contact with his mother and with objects from the first phase onward. This is also evident in the multimodal transcription presented in Chart 1.

These findings highlight that R presents a sensory profile of hypersensitivity to visual, auditory, and tactile stimuli, which hinders his engagement in pleasurable interactions with his mother. This aspect is noted as a sensory-motor characteristic of infants who later develop ASD^(15)^. Since R’s mother assumes a subject and a speaker in her child^(6)^, she seeks to interpret his signals, showing that she invests in the dialogue, much like B's mother. However, the multimodal transcriptions show that R's mother struggles to validate her child’s feelings, often assigning directive interpretations to his expressions. This may be related both to her difficulty in perceiving the origin of her child’s discomfort and to R’s limited time-window for gesture-speech synchronization, meaning his abilities to synchronize and express himself did not allow sufficient space for his mother’s interpretation. As a result, she follows her own discourse, missing some of R’s manifestations, particularly in age ranges 4 and 5.

Furthermore, R's repertoire is restricted; although he performs several pantomimes (age range 5), they are always the same, characterized by repetitive behaviors consistent with an ASD diagnosis. It can be stated that R’s mother initiated the processes of homology and interpretance^(6)^ necessary for establishing the conjunctive relations that form the basis of the first enunciative mechanism of language acquisition^(4)^. However, R’s difficulties with engagement, shared attention^(1,2)^, and the presence of significant discomfort hindered the maintenance of initial protoconversations in this dyad.

In contrast, in B’s case, the gesture-speech synchrony allows the mother to form broader interpretive hypotheses, consolidating from the early stages the conjunctive relations^(4)^ in which B participates with vocalizations, smiles, and gestures, and the mother responds through her speech interpreting the child’s expressions^(5)^. B demonstrates the ability to integrate gesture and speech in a way that allows the mother to recognize him as a speaker^(7)^. This recognition enables the mother to gradually reduce physical contact in the later phases and to synchronize more seamlessly with her child. Overall, her interpretations align with B’s manifestations. B’s productions are not left adrift, and thus the transition from shown reference to spoken reference emerges clearly by the fourth age range, revealing his capacity for coreference^(4)^. In addition, B’s gestural repertoire includes a wider variety of gestures (metaphorical, emblematic, and varied pantomimes), which supports the idea that his gestures are integrated into speech, as seen in studies on the relationship between gesture and fluency in child language acquisition^(1,2)^.

Therefore, although R’s mother invests in the relationship and remains more talkative and uses more gestures to engage her baby than B’s mother, she is not successful in establishing conjunctive relations^(4)^ with R because his sensory profile seems to trigger avoidance of eye contact and maternal touch, which disrupt the synchrony between mother and child. Interestingly, maternal touch may be perceived as painful by R, stripping it of the deictic quality that could help build shared reference between mother and child. In contrast, the pain felt by R may strip the gesture of its representative character and deprive it of linguistic representation. In B’s case, this process is not hindered, as he seems to experience pleasure and registers maternal touch as a deictic indication, where words can be inscribed within this representation^(1,2)^.

In this regard, an essential condition to be analyzed in maternal behaviors in response to infant manifestations—crucial for establishing conjunctive relations^(4)^—is the mother's ability to offer an interpretation and validate the child’s feelings, as this has the effect of recognizing the child as both subject and speaker^(6)^. On the child's side, it is necessary that they can register the mother’s gestures and touch as forms of representation within language functioning. When the child experiences pain and discomfort, as was often the case with R, it becomes difficult to extract the sensorimotor invariants necessary to create stable representations of self and the world, as proposed by Bullinger^(15)^. On the contrary, pain throws R into an avoidance circuit of interaction with the other, which hinders the construction of representations necessary for the emergence of semiotic function.

Without shared attention, one of the early prerequisites for communication^(2)^, it is difficult to establish the conjunctive relations proposed by Silva^(4)^. Shared attention, as a capacity for coordinated attention with another person, is essential for social interactions because it helps understand the world, enables shared meaning-making, allows the interpretation of others, and fosters reciprocal affective synchrony. Gestural expressions also involve intentions, behavioral dispositions, and the experiential dimension of affects. The mothers of both children, in the two cases studied, interpreted their children’s manifestations based on their own experiences. In B’s case, the mother found meaning in the child’s gestural reactions because the context allowed her to interpret his expressions accurately. In R’s case, however, the mother, based on her own knowledge, struggled to achieve affective synchrony due to the absence of sensory experiences similar to those of her child^(16)^.

It is known that gestures include hand movements, eye gaze, facial expressions, and head movements^(1,2)^. More gestures were observed in the mothers than in the babies. Facial expressions convey people’s emotional states and help the interlocutor interpret paths to either promote or avoid interpersonal interaction. Universal emotions such as disgust, fear, joy, surprise, and sadness reveal facial characteristics based on physiologically rooted emotions, which are not learned but form a basis for composing more complex gestures^(16)^. When these expressions are not understood, as occurred with R, it becomes difficult to sustain a place of enunciation^(6)^, especially for a child whose production is anchored in gesturality.

Another fundamental aspect to consider, from the multimodal perspective of language acquisition^(1,2)^, is that gesture is a modality of language and therefore belongs to the verbal domain. Gesture not only accompanies speech but also integrates with it and is fundamental for the infant to occupy their place of enunciation^(6)^. It is clear that the more gestures B produces, the more he also progresses from shown to spoken reference. Thus, gesture can be viewed as a semiotic system that integrates the semiotic domain and is fundamental as the first form of meaning-making within the I-YOU language functioning^(4)^. Gesture-speech synchrony lays the foundation for the baby to synchronize with the mother in language functioning, allowing the infant to occupy and maintain a place of enunciation^(7)^.

Upon completing the analysis in this study, it becomes clear that there is a mismatch in the language functioning between R and his mother, and a progressively strengthened alignment between B and his mother. The data also highlight the importance of timely interventions so that mothers of children at risk of evolving towards an ASD diagnosis can understand how to compensate for their children's sensory difficulties, thereby enabling communication. This understanding is essential for sustaining a place of enunciation for children experiencing psychic suffering and for minimizing the symptoms that emerge as signs of crystallized suffering around the second year of life, including language disorder. Speech-language pathologists who know how to observe gesture-speech synchrony between mothers and babies can use this knowledge to build individualized therapeutic plans through timely interventions.

Although this case study is limited in its generalizability to the broader ASD population, it offers insights for future research that incorporates gestures as an important aspect of childhood speech fluency and therefore also fundamental in speech-language research.

## CONCLUSION

Although the gestural typology of B and R is similar, the frequency, quality, and, above all, the gesture-speech synchrony differ in the language functioning of the dyads.

In B's case, without ASD, gesture-speech synchrony, eye contact, and vocalizations converge to sustain protoconversations with his mother. In R's case, with ASD, the mismatch within the dyad arises from the presence of pain and R’s difficulty in remaining attentive, engaged, and fully present in the relationship. His sensory restrictions do not create the bodily conditions necessary for him to progressively occupy his place of enunciation towards language acquisition and instead fuel the disconnection between him and his mother.

## Footnotes

* The original name of the script is risk but it was recently replaced by reference.
