# Supplementary material for: Comparison of gesture-vocal synchrony and gestures of two children aged 3 to 26 months with and without autism spectrum disorder
Source: Codas. 2025 Oct 27;37(5):e20240311. doi: 10.1590/2317-1782/e20240311en (PMC12617283; doi:10.1590/2317-1782/e20240311en)
Supplement: Translation

# Translation: Comparação da sincronia gesto-vocal e gestualidade de duas crianças de 3 a 26 meses com e sem transtorno do espectro autista

**DOI:** 10.1590/2317-1782/e20240311pt

**Authors:** Gabriela Luisa Gantier Fernández, Marianne Carvalho Bezerra Cavalcante, Ana Paula Ramos de Souza

**Affiliations:** 1 Universidade Federal de Santa Maria – UFSM - Santa Maria (RS), Brasil.; 2 Programa de Pós-graduação em Letras, Universidade Federal da Paraíba – UFPB - João Pessoa (PB), Brasil.; 3 Departamento de Saúde e Comunicação Humana, Universidade Federal do Rio Grande do Sul – UFRGS - Porto Alegre (RS), Brasil.; 4 Programa de Pós-graduação em Distúrbios da Comunicação Humana – PPGDCH, Universidade Federal de Santa Maria – UFSM - Santa Maria (RS), Brasil.

**Keywords:** Autismo, Fala, Multimodalidade, Linguagem, Desenvolvimento Infantil

## Abstract

O objetivo foi comparar a sincronia gesto-vocal no funcionamento de linguagem entre mães e bebês e a tipologia gestual de bebês dos 3 aos 26 meses, sendo um deles com transtorno do espectro do autismo (caso R), diagnosticado aos três anos, e outro sem diagnóstico (caso B). A partir de um banco de filmagens das interações da mãe e do bebê realizadas dos 3 aos 26 meses, foram selecionados momentos em que houve maior interação entre mãe e bebê. Foram analisados por meio do software Eudico Linguistic Annotator (ELAN), considerando categorias multimodais de linguagem gestual e de fala das mães e dos bebês para análise estatística descritiva. Os resultados evidenciaram diferenças nas frequências e tipos gestuais entre os bebês e também na sincronia entre eles e suas mães. No caso de B. a sincronia gesto-vocal e variedade de gestos está em um contexto de conjunção entre ele e sua mãe no primeiro ano de vida, o que fez emergir o segundo mecanismo enunciativo no segundo ano de vida. No caso R, as relações de conjunção não se estabeleceram, pois as dificuldades na leitura de gestos de desconforto e incômodo pela mãe, muito presentes no bebê desde seus primeiros meses de vida, dificultaram o engajamento e a atenção compartilhada da mãe e seu bebê, desfavorecendo a inscrição do gesto como linguagem. R. apresentou atraso na aquisição da linguagem aos 26 meses. Apesar da tipologia gestual de B e R serem similares, as frequências, a qualidade e, sobretudo, a sincronia gesto-vocal diferem entre as díades.

## INTRODUÇÃO

A sincronia gesto-vocal tem sido estudada na compreensão da emergência da fluência infantil^(1,2)^, evidenciando, a partir de trabalhos clássicos sobre a gestualidade humana, que há uma continuidade e integração entre gesto e fala no funcionamento da linguagem infantil. Neste sentido, a sincronia se dá quando as modalidades de uso da língua (fala e gesto) coatuam na produção linguística, promovendo pontos de saliência no enunciado. Trata-se de uma abordagem que traz contribuições para pensar o desenvolvimento atípico da linguagem^(3)^.

Neste trabalho, incorpora-se esta abordagem multimodal das interações linguísticas à perspectiva enunciativa de aquisição da linguagem proposta por Silva^(4)^ para pensar as relações de conjunção e disjunção iniciais entre mãe e bebê, permeadas por processos de homologia e interpretância na relação entre manifestações gestuais e vocálicas do bebê e verbais da mãe, presentes nas protoconversações iniciais^(5)^.

Sabe-se que a passagem da dependência discursiva do bebê em relação à mãe, presente nas relações conjuntivas, para o reconhecimento dos efeitos de suas manifestações no outro, presente nas relações disjuntivas, impulsionam os bebês para a constituição do segundo mecanismo enunciativo, que é a passagem da referência mostrada à falada^(4)^. Para que esta passagem se dê é preciso que o bebê tenha condições de ocupar seu lugar de enunciação e a mãe de sustentar este lugar para ele^(6)^. Nesse sentido, um estudo^(7)^ evidenciou tanto dificuldades no exercício das funções parentais, na suposição de sujeito e no estabelecimento da demanda^(7)^, quanto risco para evolução para um quadro de TEA^(7)^. Oliveira et al.^(8)^ observaram maior correlação entre roteiros de sofrimento psíquico no primeiro semestre de vida. No segundo, terceiro e quarto semestres de vida dos bebês, o sofrimento psíquico e o atraso na aquisição da linguagem coincidem, mas também há casos de atraso na aquisição da linguagem sem risco psíquico. Esses estudos mostraram que a presença de sofrimento psíquico, seja ele na direção da estruturação de autismo ou não, correlaciona-se de modo significativo com presença de atraso na aquisição da linguagem, analisado em uma perspectiva enunciativa.

A forma de avaliação em ambos estudos^(7,8)^ utilizou os Sinais Enunciativos de Aquisição da Linguagem^(9,10)^ que analisam relações conjuntivas e disjuntivas do primeiro mecanismo enunciativo e também a emergência do segundo mecanismo enunciativo, mas sem uma discriminação mais fina dos aspectos gestuais. Por isso, nesta pesquisa, buscou-se investigar não apenas os efeitos enunciativos do sofrimento psíquico, como também agregar ao estudo um olhar multimodal do processo de aquisição da linguagem, sobretudo em relação ao primeiro e segundo mecanismo enunciativo^(4)^. Isso porque se acredita que a investigação das características multimodais de sincronia gesto-vocal, como a fala, o gesto, o olhar endereçado, as expressões faciais, os movimentos da cabeça e outros indicadores pode elucidar sinais para a detecção precoce de risco, e para a intervenção oportuna que busca o estabelecimento das relações enunciativas de conjunção mãe-bebê, que são a base para a emergência dos demais mecanismos enunciativos no processo de aquisição da linguagem.

Considerando o grupo de crianças com atraso na aquisição da linguagem, embora já haja estudos da sincronia gesto-vocal em crianças com síndrome de Down^(3)^, não se observaram estudos dessa sincronia em crianças com TEA na realidade nacional de modo integrado à perspectiva enunciativa. Assim, os objetivos deste estudo são comparar a sincronia gesto-vocal no funcionamento de linguagem entre mães e bebês, e a tipologia gestual de bebês dos 3 aos 26 meses de idade, sendo um deles com transtorno do espectro do autismo (caso R) com diagnóstico aos 3 anos e outro sem diagnóstico (caso B).

## MÉTODO

Trata-se de uma pesquisa qualitativa longitudinal de estudos de casos, observacional, analítica e comparativa; de duas crianças, uma com TEA (R) e outra sem (B), acompanhadas dos 3 meses aos 26 meses. A pesquisa foi aprovada no comitê de ética em pesquisa da universidade sobre número de CAAE 28586914.0.0000.5346, número do parecer 1.929.266. Os responsáveis pelas crianças assinaram o termo de consentimento livre e esclarecido, autorizando a pesquisa.

Os dados de filmagens das interações entre os bebês e suas mães analisados neste estudo, provém de um banco de dados maior em que os bebês foram filmados em seis faixas etárias por 15 minutos. Além disso, os bebês e suas mães foram avaliados pelos Sinais enunciativos de aquisição da linguagem (SEAL)^(9,10)^ pelos Indicadores clínicos de referência/risco^^*^^ ao desenvolvimento infantil (IRDI)^(11)^, Sinais PREAUT^(12)^, MCHAT^(13)^ e Escala Bayley III^(14)^. Os seguintes procedimentos de coleta e avaliação estão descritos para cada faixa etária:

- Faixa 1 – 3 meses e um dia a 4 meses e 29 dias: Na posição sentada em um bebê-conforto (9 minutos). Nesta posição, a mãe foi convidada a cantar (3 minutos), falar (3 minutos) e oferecer um objeto ao bebê - um cachorro de borracha sem barulho (3 minutos). Nas posições prono (3 minutos) e supino (3 minutos), podendo ou não a mãe oferecer um brinquedo ou conversar com o bebê. IRDI faixa I e Sinais PREAUT, SEAL.
- Faixa 2- 5 meses e um dia a 6 meses e 29 dias: Na posição sentada em um bebê-conforto (9 minutos). Nesta posição, a mãe foi convidada a cantar (3 minutos), falar (3 minutos) e oferecer um objeto ao bebê - um cachorro de borracha sem barulho (3 minutos). Nas posições prono (3 minutos) e supino (3 minutos), podendo ou não a mãe oferecer um brinquedo ou conversar com o bebê. SEAL.
- Faixa 3-8 meses e um dia a 9 meses e 29 dias: sentada sem apoio se possível (9 minutos). Na posição sentada, orientou-se a mãe que cantasse ao bebê por 3 minutos, conversasse por 3 minutos e que oferecesse um objeto ao bebê (o cachorro de borracha) por mais 3 minutos. Nas posições prono (3 minutos) e supino (3 minutos), a mãe foi orientada a estimular o bebê a rolar e rastejar/engatinhar e também foi observada a tentativa ou não do bebê de se levantar, com ou sem apoio. IRDI fase II, Sinais PREAUT, SEAL.
- Faixa 4- 11 meses e um dia a 12 meses e 29 dias: Nesta etapa, o bebê ficou livre para explorar uma caixa de brinquedos temáticos (transportes, comidinha, boneca, animais) com a mãe sobre o tapete de EVA. Ele poderia ficar sentado ou andar desde que permanecesse sobre o tapete para facilitar a filmagem. Foram 10 minutos com a mãe e 5 minutos com a entrada do terapeuta para verificar alguns sinais enunciativos. IRDI fase III, SEAL.
- Faixa 5- 17 meses e um dia a 18 meses e 29 dias: mesmo procedimento da fase 4. IRDI fase IV, SEAL, BAYLEY III, MCHAT.
- Faixa 6- 22 meses e um dia a 26 meses: mesmo procedimento das fases 4 e 5. SEAL, BAYLEY III, MCHAT.

Do banco de filmagens que incluíam momentos em que as mães cantavam, conversavam e brincavam com as crianças, foram selecionados momentos em que as interações face a face estiveram mais presentes, padronizando um tempo e condições semelhantes entre os dois casos, conforme se pode visualizar na Tabela 1.

**Tabela 1:** Descrição das faixas etárias e tempo de análise de filmagens por sujeito

Nº	FAIXA ETÁRIA	Sujeito	Idade de cada criança na coleta	Constituição temporal das amostras	
				Tempo original do vídeo	Amostra selecionada para análise ELAN
**1**	3m 1dia – 4m 29d.	R	3 m 25d	18min25 seg	2 min 44 seg
		B	3m7d	14min 19 seg	3 min 31 seg
**2**	5m 1d – 7m 29d	R	6 m 7d.	19 min. 35 seg	2 min 42seg
		B	6m 6d	21 min 51 seg	2 min 32 seg
**3**	8m 1d- 9 m 29 d	R	8 m 8 d	16min 1seg	2min 33 seg
		B	9m 29d	12 min 31 seg	2 min 22 seg
**4**	11m 1d- 12m 29d	R	12m 17d	17 min 17seg	2min 40 seg
		B	12m13d	16min 58 seg	2 min 38 seg
**5**	17m 1d – 18m 29 d.	R	17 m.	19min 34 seg	2 min 43 seg
		B	17m 19d	13 min 9 seg	2 min 47 seg
**6**	22m 1d – 26 m 29d	R	26 m 20 d	16min 3 seg	2min. 53 seg
		B	22m	14 min 29 seg	2min 31 seg

Cabe destacar que identificados os sinais de risco em R. durante as primeiras avaliações, buscou-se oferecer uma intervenção oportuna mas ele permaneceu em terapia por apenas dois meses, dos 10 aos 12 meses, o que teve efeitos na fase 5 mas não se sustentou no tempo pois aos 26 meses, sua última filmagem, estava com sinais claros de TEA.

A partir desta seleção, os dados foram analisados por meio do software Eudico Linguistic Annotator, (ELAN), o que possibilitou a transcrição de recortes de momentos de interação chave para visualização detalhada, marcação de tempos e descrições segundo as trilhas estabelecidas conforme descrito na Tabela 2.

**Tabela 2:** Normas das transcrições multimodais

Trilhas	Símbolos
Fala mãe	-.......................-
Silêncio da mãe	*.......................*
Movimento da cabeça da mãe	/......................./
Olhar da mãe	(.......................)
Movimento mãos da mãe	_......................._
Gestos Mãe	+.......................+
Fala Criança	--.......................--
Silêncio criança	**.......................**
Movimento de cabeça da criança	//.......................//
Olhar da criança	((.......................))
Movimento mãos da criança	__......................__
Gestos criança	++......................++

Também se buscou identificar a tipologia gestual emergente em cada caso a cada faixa etária conforme descrito na Tabela 3.

**Tabela 3:** Tipologia gestual

Pausas Vocais	Foram considerados os silêncios maiores do 7 seg de duração como pausas vocais.
Tipologia gestual	Foi analisada a tipologia gestual corporal, considerando emblemas, pantomimas, gestos de desconforto, gestos de felicidade, gestos preenchedores, entre outros que emergiram.
Troca de Olhares	O contato visual foi analisado em segundos, e também analisou-se o endereçamento da mãe e da criança.
Tempos das trocas	Os tempos de troca sincrônica nas díades foram marcados.
Toque das mães	O toque das mães pertence aos gestos dêiticos e algumas vezes exercem papel como preenchedores dentro da tipologia, mas foi analisado separadamente da tipologia para a melhor compreensão sensorial nos casos.

A partir da anotação no ELAN foram produzidas estatísticas descritivas que são apresentadas nos resultados.

## RESULTADOS

Na Tabela 4 são descritos os resultados de categorias gerais das análises.

**Tabela 4:** Descrição geral dos resultados

Faixas etárias	Casos	Silêncios mães	Gestos mães	Fala das mães	Olhar mães	Silêncio crianças	Fala criança	Contato visual díade	Sincronia gesto vocal criança
1	R	0	9	60	31	6	24	6	7
	B	0	27	63	23	5	49	10	8
2	R	0	15	54	17	6	5	3	0
	B	0	4	52	33	4	27	11	4
3	R	0	26	59	14	4	18	3	12
	B	0	13	48	9	4	31	6	12
4	R	0	27	50	15	8	17	5	2
	B	0	20	77	17	9	26	11	7
5	R	2	11	54	27	6	11	4	5
	B	0	15	60	27	2	39	12	26
6	R	0	8	69	30	8	15	1	8
	B	0	21	48	36	1	32	15	20

Esses resultados evidenciam que não há distinções importantes entre as mães. Já entre as crianças há maiores distinções no contato visual, fala da criança e sincronia gesto-vocal da criança, em que os valores do caso R sempre são menores. Essas distinções ficam mais visualizáveis nas Figuras 1 e 2.

**Figura 1:** Análise da evolução dos silêncios e vocalizações

**Figura 2:** Análise da evolução do contato visual e sincronia gesto-vocalização

Portanto, enquanto B amplia suas vocalizações rumo à fala, a sincronia gesto-vocal e o contato visual com sua mãe, diminuindo o silêncio, R mantém a vocalização e a sincronia gesto-vocal em patamares mais baixos e diminui o contato visual com sua mãe, aumentando seus períodos de silêncio. Deste modo, pode-se dizer que, enquanto a díade B consolida o funcionamento da linguagem em diálogo, a díade R não evolui como esperado no decorrer do acompanhamento.

Em relação à evolução gestual na Tabela 5 estão as expressões gerais e na Figura 3 a tipologia gestual de cada criança, considerando a classificação inicial de gestos adotadas na pesquisa.

**Tabela 5:** Evolução das expressões gerais dos bebês

expressão	R1	B1	R2	B2	R3	B3	R4	B4	R5	B5	R6	B6
sorriso/felicidade	0	6	0	10	8	8	4	0	0	1	6	1
queixa/nervoso	3	0	0	0	0	0	1	0	0	0	0	0
incômodo/desconforto	2	0	3	0	9	0	2	0	1	0	2	0
susto/defesa	3	0	0	0	0	0	0	0	2	0	0	0
tristeza	0	0	2	0	1	0	0	0	0	0	0	0

1, 2, 3, 4, 5, 6= número da faixa etária

**Figura 3:** Evolução gestual dos bebês

No caso de R, existiam expressões faciais gerais (Tabela 5), manifestando gestos de queixa, incômodo, susto, de nervoso, que diferiam de B desde a primeira faixa. Na faixa 2, queixa e incômodo dominam suas manifestações. Já, nas faixas 3 e 4, acrescentaram-se aos gestos de queixa, incômodo e desconforto, gestos de felicidade (Tabela 5), pantomima, dêitico e emblema (Figura 3). Isso demonstra que ele pode ter uma tipologia similar a B na variedade esperada de gestos, mas difere dele na presença constante de incômodo, desconforto e queixa. Outra diferença está na qualidade dos gestos, pois a pantomima de R era sempre a mesma relativa à reprodução de bum-bum para o carrinho, demonstrando seu hiperfoco no carrinho, enquanto as de B variavam, abrangendo diferentes animais, transportes e cenas de alimentação.

No Quadro 1 estão exemplos da transcrição multimodal da fase 1 e 6 de ambos casos.

**Quadro 1:** exemplos de transcrições multimodais de B e R

Transcrições multimodais	
**Faixa 1 caso R**	**Faixa 1 caso B**
MR-anda brinca um pouquinho ehh, ta com preguiça?	MB-nhammmm-
(olha para o pé olha para perna )	/Deixa cabeça em posição firme /
(Olha para a criança)	_leva as mãos para trás e faz movimentos no ar_
	+gesto pantomima de aranha caminhando+
	(olha para os pés da criança)
R//leva a cabeça para esquerda//	B--Vocalização com vogal aaaa--
((olha para outro ponto na esquerda))	((olha para a mãe sorrindo e tendo prazer com o contato ))
	
MR_abaixa as mãos_ (Olha para baixo)	MB-Eu vou pegar o bebezinho-
-cadê o bebê que grita ahh?	_pega os pés da criança e acaricia_
(olha para a criança)	(olha para a face da criança)
**Faixa 6 caso R**	**Faixa 6 caso B**
R((olha para os outros brinquedos no chão))	B --“gr ahhhh u gr ahh u” onomatopeia dinossauro--
++faz gesto de reação de dor++	++ gesto pantomima de dinossauro atacando++
Sincronia fala-gesto criança	Sincronia gesto-vocal
__toca com a mão esquerda o chão__	
MR_com a outra mão, apresenta o brinquedo e dá para a criança_	MB_faz balançar a boneca no chão_
R((fecha os olhos))	B__move rapidamente dinossauro no ar__
	((olha para os brinquedos no chão))

R: caso R, MR: mãe de R, Caso B, MB: mãe de B

Observa-se, no Quadro 1, mais desconforto e menos evolução na atenção compartilhada e engajamento com sua mãe por parte de R, quando comparado a B. Enquanto B. demonstra prazer no contato físico, R. evita o contato com a mãe na fase 1. Na fase 6, B. está dramatizando um ataque com dinossauro a uma boneca com a mãe valendo-se de pantomima, e R. praticamente não se engaja nas propostas de brincadeira de sua mãe.

Cabe destacar que nos momentos de desconforto de R. era comum a mãe interpretar sua reação como se estivesse bravoe não como um sinal de dor. Durante os dois anos de acompanhamento a mãe não conseguia perceber que R. tinha dor física ligada às questões sensoriais, relacionadas à hipersensibilidade tátil, auditiva, visual e vestibular, entre outras dificuldades.

## DISCUSSÃO

Desde a primeira fase, é clara a diferença entre R e B quanto ao fato de que, nas transcrições multimodais, R apresenta gestualidade menos variada e com menor frequência e tempo. Destaca-se a menor sincronia entre fala e gesto e contato visual, como se viu na Figura 2. Além disso, R possui menor quantidade de fala do que B, e mais momentos de silêncio observáveis na Figura 1. Há muitos momentos de desconforto e incômodo de R enquanto B sempre está sorridente e desfrutando do contato físico, visual e corporal com a mãe e os objetos desde a fase 1. Isso pode ser também visto na transcrição multimodal apresentada no Quadro 1.

Esses dados colocam em relevo que R possui um perfil sensorial de hipersensibilidade visual, ao som e ao toque que dificulta engajar-se em relações prazerosas com a mãe. Esse aspecto é ressaltado como uma característica sensório-motora de bebês que desenvolvem TEA em idades mais avançadas^(15)^. Como supõe um sujeito e um falante^(6)^ em R., a mãe busca uma interpretação ao que o filho está trazendo, demonstrando que investe no diálogo, tanto como a mãe de B. Nas transcrições multimodais, observa-se que a mãe de R tem dificuldade de validar os sentimentos do filho, atribuindo interpretações diretivas às suas manifestações, o que pode estar relacionado à dificuldade de perceber a origem do desprazer do filho, por um lado, e à dificuldade originária do tempo de sincronização gesto-fala ser pequeno para R, ou seja, as habilidades de R para sincronizar e se manifestar não permitiam o espaço necessário para a interpretação da mãe. Por isso, ela segue um discurso próprio que não aproveita algumas das manifestações de R, sobretudo nas faixas etárias 4 e 5. Além disso, o repertório de R é restrito, pois, mesmo fazendo várias pantomimas (Faixa 5), elas eram sempre as mesmas, caracterizando os comportamentos repetitivos previstos no diagnóstico de TEA. Pode-se afirmar que a mãe de R. colocava em curso os processos de homologia e intrepretância^(6)^ necessários ao estabelecimento das relações de conjunção necessárias ao primeiro mecanismo enunciativo de aquisição da linguagem^(4)^. No entanto, a dificuldades de engajamento e atenção compartilhada^(1,2)^ de R., além da dor e desconforto muito presentes, dificultavam a manutenção das protoconversações iniciais nesta díade.

Já no caso de B, a sincronia gesto-fala em suas manifestações parece permitir hipóteses interpretativas mais amplas da mãe e a consolidação desde as primeiras etapas das relações conjuntivas^(4)^ em que B comparece com vocalizações, sorrisos e gestos, e a mãe com sua fala interpretando^(5)^ as manifestações do filho. B. possui as condições de integrar gesto e fala de modo a ser reconhecido como falante^(7)^ pela mãe. Esse reconhecimento faz com que sua mãe não necessite recorrer tanto ao contato físico nas últimas etapas e consiga encontrar-se de modo sincrônico com o filho. Em geral, suas interpretações estão em acordo com as manifestações de B. As produções de B não ficam à deriva e, com isso, a passagem da referência mostrada à falada para B emerge com nitidez já na quarta faixa etária, evidenciando sua capacidade de correferir^(4)^. Além disso, a gestualidade de B inclui maior variedade de gestos (metafóricos, emblemas e pantomimas variadas). Isso permite afirmar que sua gestualidade está integrada à fala conforme preveem estudos em aquisição que analisam a relação entre gestualidade e fluência da fala infantil^(1,2)^.

Portanto, embora a mãe de R invista na relação e mantenha-se mais falante e com mais gestos para captar seu bebê do que a mãe de B, ela não é bem-sucedida no processo de criação de relações conjuntivas^(4)^ com R porque seu perfil sensorial parece produzir evitação do contato visual e do toque da mãe que quebram a sintonia entre mãe e filho. É interessante observar que o toque da mãe pode ser sentido como dor e isso lhe retira o caráter dêitico que poderia ter na construção da correferência mostrada entre mãe e filho. Ao contrário, a dor sentida por R pode retirar o caráter representativo do gesto e desprover-lhe da representação linguística. Já, no caso de B, esse processo não fica impedido porque ele parece sentir prazer e registrar o toque da mãe como uma indicação dêitica^(1,2)^ do corpo e junto a ele a palavra pode se inscrever nessa representação.

Nesse sentido, uma das condições importantes de se analisar nos comportamentos maternos frente às manifestações dos bebês, que é fundamental para que se estabeleçam as relações conjuntivas^(4)^, é que a mãe possa oferecer uma interpretação e validar os sentimentos das crianças, pois isso tem um efeito de reconhecimento da criança com sujeito e falante^(6)^. Do lado da criança, que ela possa registrar a gestualidade e o toque maternos como formas de representação em um funcionamento de linguagem. Se a criança sente dor e desconforto, como foi comum observar em R, fica difícil ele extrair as invariantes sensório-motoras para criar representações estáveis de si e do mundo, como prevê Bullinger^(15)^. Ao contrário, a dor lança R em um circuito de evitamento da interação com o outro e, por isso, com dificuldades na construção das representações necessárias à emergência da função semiótica.

Sem atenção compartilhada, um dos requisitos iniciais para a comunicação^(2)^, há dificuldade de se produzirem as relações conjuntivas previstas por Silva^(4)^. A atenção compartilhada, ao ser uma capacidade de coordenação à atenção com o outro, é uma habilidade essencial para as interações sociais, porque ajuda a entender o mundo, ter produção compartilhada de sentido, entender e interpretar as pessoas e entrar em sintonia afetiva recíproca. Também estão envolvidos nas expressões gestuais intenções, disposições de comportamentos, uma dimensão de experiência dos afetos. As mães das crianças, nos dois casos estudados, interpretam as manifestações das crianças baseadas nas suas experiências. No caso de B, a mãe encontra sentido nas reações gestuais da criança porque o contexto faz com que ela interprete adequadamente suas expressões gestuais. No caso de R, no entanto, a mãe, a partir dos seus conhecimentos, apresenta maior dificuldade de entrar em uma sincronia afetiva por ausência de experiências sensoriais similares à de sua criança^(16)^.

Sabe-se que o gesto inclui movimentos das mãos, troca de olhares, expressão facial e movimento de cabeça^(1,2)^. Observa-se maior quantidade de gestos nas mães do que nos bebês. As expressões faciais demonstram o estado emocional das pessoas e ajudam o alocutário a interpretar caminhos para promover ou evitar a interação interpessoal. As emoções universais mostram a existência de nojo, asco, medo, alegria, surpresa e tristeza. São características faciais definidas a partir de emoções que têm origem fisiológica, não são apreendidas, mas conformam uma base para a composição de gestos mais complexos^(16)^. Se essas expressões não são compreendidas, como ocorria com R, há dificuldades na sustentação de um lugar de enunciação^(6)^, sobretudo para uma criança que ancora sua produção na gestualidade.

Outro aspecto fundamental é pensar, a partir da perspectiva multimodal de aquisição^(1,2)^, que o gesto é uma modalidade de linguagem e, portanto, está no âmbito do verbal. O gesto não só acompanha a fala, mas também a integra e é fundamental na ocupação pelo bebê de seu lugar de enunciação^(6)^. Fica claro que, quanto mais gestos B produz, mais ele também passa da referência mostrada à falada. Deste modo, pode-se pensar no gesto como um sistema semiótico que integra o domínio semiótico e que é fundamental como primeira forma de semantização no funcionamento de linguagem na dupla EU-TU^(4)^. A sincronia gesto-vocal dá as bases para que o bebê entre em sincronia no funcionamento de linguagem com sua mãe, o que lhe permite ocupar e ter sustentado um lugar de enunciação^(7)^.

Ao finalizar as análises desta pesquisa, fica claro o desencontro no funcionamento de linguagem entre R e sua mãe e o encontro cada vez mais fortalecido entre B e sua mãe. Os dados evidenciam também a importância das intervenções oportunas para que as mães das crianças que possuem risco de evoluírem para um quadro de TEA possam entender como compensar dificuldades sensoriais dos filhos e, deste modo, viabilizar a comunicação com eles. Essa compreensão é fundamental para que se sustente um lugar de enunciação para a criança em sofrimento psíquico e se produza a minimização dos sintomas que emergem como sinais de sofrimento já cristalizado ao redor do segundo ano de vida, entre os quais o distúrbio de linguagem. O fonoaudiólogo que souber observar a sincronia gesto-fala entre mães e bebês poderá tirar proveito deste conhecimento para a construção de projetos terapêuticos singulares em intervenções oportunas.

Embora esse estudo de casos seja limitado em sua generalização para a população com TEA, ele dá indicações de pesquisas futuras que incorporem a gestualidade como um aspecto importante da fluência infantil na linguagem e, portanto, também fundamental na pesquisa fonoaudiológica.

## CONCLUSÃO

Apesar da tipologia gestual de B e R serem similares, as frequências, a qualidade e, sobretudo, a sincronia gesto-vocal difere no funcionamento de linguagem das díades.

Enquanto no caso de B., sem TEA, a sincronia gesto-vocal, o contato visual, as vocalizações convergem para a manutenção das protoconversações com sua mãe, no caso de R., com TEA, o desencontro na díade se dá pela presença da dor e da dificuldade de R. manter-se atento, engajado e pleno na relação. Suas restrições sensoriais não criam as condições corporais para que pudesse ocupar de modo crescente seu lugar de enunciação rumo à aquisição da linguagem e alimentam o desencontro entre ele e sua mãe.

## Footnotes

* inicialmente eram chamados risco mas passaram a ser designados como de referência pelos autores mais recentemente.
